# Meteorological Table

**Published:** 1812-08

**Authors:** 


### METEOROLOGICAL TABLE:

*From May 26, to June 26, 1812.*

| | D. | Therm. | | | Barom. | | Hygrom. *Dry.* | | | *Damp.* | | | Winds. | Atmos. Variation. |
|---|---|---|---|---|---|---|---|---|---|---|---|---|---|---|
| | | 65 | 73 | 61 | $29^{6}$ | — | — | 35 | 15 | 27 | — | — | W.. | F... |
| | 28 | 58 | 70 | 60 | $29^{7}$ | $^{6}$ | 10 | 19 | 2 | | | | S.. | F... R... |
| | 29 | 63 | 73 | 57 | $29^{6}$ | $^{7}$ | | | | 3 | 25 | 5 | S.. | F... R... |
| | 30 | 58 | 67 | 58 | $29^{8}$ | — | — | 20 | 16 | 1 | — | — | SW.. | F.... |
| | 31 | 57 | 69 | 55 | $29^{8}$ | — | 8 | 34 | 6 | | | | SW... | F.... R.. |
| | 1 | 55 | 56 | 54 | $29^{8}$ | $^{9}$ | | | | 3 | 11 | 7 | SW... | R... F.... |
| ◑ | 2 | 52 | 65 | 60 | 30 | — | — | 10 | 1 | 8 | — | — | W.. | C.. F.... |
| | 3 | 60 | 62 | 60 | 30 | — | | | | 3 | 8 | 7 | NW. | R. C... F.... |
| | 4 | 57 | 76 | 62 | $30^{1}$ | — | — | 20 | 15 | 6 | — | — | N..E. | F.... C.. |
| | 5 | 59 | 72 | 52 | $30^{1}$ | — | 7 | 30 | 15 | | | | E.. | F.... C.. |
| | 6 | 55 | 70 | 56 | $30^{1}$ | $^{2}$ | 9 | 30 | 15 | | | | SE. | C.... F.... |
| | 7 | 54 | 74 | 60 | $30^{2}$ | $^{3}$ | 9 | 30 | 20 | | | | NE.. | F.... |
| | 8 | 56 | 68 | 55 | $30^{3}$ | $^{4}$ | 10 | 14 | 11 | | | | E..NE.. | C... F.... |
| ● | 9 | 55 | 65 | 57 | $30^{4}$ | $^{2}$ | 9 | 20 | 11 | | | | W.. | C... F.... |
| | 10 | 57 | 63 | 55 | $30^{2}$ | — | 13 | 20 | 11 | | | | NE.. | F..C...F.... |
| | 11 | 53 | 79 | 65 | $30^{2}$ | — | 10 | 20 | 22 | | | | W..NW.. | F.... C... |
| | 12 | 60 | 79 | 65 | 30 | — | 11 | 25 | 13 | | | | NW.. | C... F.... |
| | 13 | 58 | 76 | 57 | $29^{9}$ | — | 12 | 22 | 14 | | | | W.. | C.. F.... |
| | 14 | 57 | 73 | 59 | $29^{9}$ | [illegible] | 11 | 27 | 15 | | | | SW.. | F.... |
| | 15 | 59 | 69 | 57 | $29^{8}$ | — | 10 | 14 | 9 | | | | SW... | F.... R... |
| ◐ | 16 | 59 | 68 | 54 | $29^{8}$ | $^{7}$ | 10 | 35 | 20 | | | | W... | F.... |
| | 17 | 52 | 60 | 55 | $29^{5}$ | $^{8}$ | 11 | 9 | — | | | | S..N.. | R... F.... |
| | 18 | 54 | 59 | 56 | $29^{8}$ | $^{7}$ | 7 | — | 1 | | | | W... | F... R.... |
| | 19 | 55 | 62 | 54 | $29^{5}$ | $^{4}$ | — | 27 | 11 | 6 | — | — | SW... | R.. F... R... |
| | 20 | 55 | 59 | 53 | $29^{4}$ | [illegible] | 5 | 8 | 4 | | | | W... | F... R... F.... |
| | 21 | 55 | 60 | 55 | $29^{6}$ | $^{7}$ | 1 | 11 | 8 | | | | W... | F... R.. F.... |
| | 22 | 53 | 60 | 53 | $29^{7}$ | — | 3 | 13 | 6 | | | | W... | F... R.. F.... |
| | 23 | 52 | 64 | 53 | $29^{8}$ | — | 6 | 40 | — | | | | W.. | F.... R.. |
| ○ | 24 | 55 | 64 | 53 | $29^{9}$ | 30 | — | 24 | 8 | | | | | F... R... |
| | 25 | 55 | 62 | 53 | 30 | $29^{8}$ | 12 | 22 | 10 | | | | | R.. F.. R.. |
| | 26 | 50 | 55 | 50 | $29^{5}$ | $^{3}$ | | | | 9 | 21 | — | | R... R... F. |

Quantity of rain from May 26th to June 26th, one inch $\frac{58}{100}$.

May 29th. Distant thunder from the west, with rain in the evening.

June 8th. Mercury in the barometer very high.

15th. Sudden and heavy storm of rain in the night.

20th. Stormy showers, with hail.

21st. Stormy showers.

Acute catarrhal affections have decreased considerably, but the most numerous cases afforded by the records of an extensive dispensary, are described as *Tussis cum Dyspnœa*, by which is meant a form of chronic catarrh. Cases of acute rheumatism have appeared, and some solitary instances of rubeola and casual small-pox.

### METEOROLOGICAL TABLE.

### *From June 26, to July 26,* 1812.

| | D. | Therm. | | | Barom. | | Hygrom. *Dry.* | | | *Damp.* | | | Winds. | Atmos. Variation. |
|---|---|---|---|---|---|---|---|---|---|---|---|---|---|---|
| | 27 | 52 | 58 | 50 | $29^9$ | — | 3 | 27 | — | — | — | 20 | | F ... R ... |
| | 28 | 52 | 60 | 51 | $29^9$ | $30^1$ | 10 | 17 | 13 | | | | NW.. | F ... |
| | 29 | 54 | 71 | 57 | 30 | $29^9$ | 10 | 12 | 10 | | | | W.. | F ... |
| | 30 | 58 | 61 | 57 | 30 | $29^9$ | 10 | 12 | 10 | | | | W.. | R .. C . R ... |
| ◑ | 1 | 57 | 62 | 55 | $29^7$ | $^5$ | | | | 5 | 2 | 3 | | R ... — ... |
| | 2 | 58 | 64 | 58 | $29^5$ | — | — | 13 | 4 | 8 | — | — | | F ... R ... |
| | 3 | 59 | 66 | 55 | $29^7$ | $^9$ | 2 | 15 | 10 | | | | | C . F ... |
| | 4 | 55 | 67 | 54 | 30 | $^1$ | 5 | 35 | 20 | | | | S.. | F .... |
| | 5 | 60 | 64 | 58 | $30^1$ | — | 15 | 20 | 13 | | | | SW... | F ... |
| | 6 | 60 | 68 | 63 | $30^1$ | $^2$ | 5 | 17 | 15 | | | | W.. | F .... |
| | 7 | 62 | 72 | 68 | $30^4$ | — | 8 | 15 | 10 | | | | W.. | F ... |
| ● | 8 | 65 | 74 | 63 | $30^4$ | — | 10 | 17 | — | | | | E.. | F .... |
| | 9 | 61 | 78 | 65 | $30^4$ | $^2$ | 14 | 45 | 27 | | | | E. | F .... |
| | 10 | 66 | 79 | 64 | $30^3$ | $^4$ | 12 | 15 | 16 | | | | NE.. | F .... |
| | 11 | 62 | 75 | 63 | $30^4$ | $^3$ | 15 | 35 | 20 | | | | W.. | F .... |
| | 12 | 61 | 68 | 58 | $30^2$ | — | 24 | 17 | 28 | | | | E. | F .. |
| | 13 | 63 | 69 | 60 | $30^2$ | — | 24 | 35 | 26 | | | | SE.. | F .... |
| | 14 | 62 | 69 | 56 | $30^2$ | — | 22 | 28 | 18 | | | | S.. | F ... R ... F ... |
| | 15 | 56 | 73 | 63 | $30^1$ | — | 17 | 49 | 30 | | | | N.. | F ... |
| ◐ | 16 | 61 | 66 | 63 | 30 | $29^9$ | 23 | 20 | 17 | | | | E.. | F ... R . F .. |
| | 17 | 64 | 67 | 64 | $29^9$ | $30^1$ | 16 | 10 | — | | | | N.. | C .. |
| | 18 | 65 | 79 | 64 | $30^1$ | — | 3 | 35 | 15 | | | | N.. | F ... |
| | 19 | 64 | 71 | 65 | 30 | $29^7$ | 10 | 10 | — | — | — | 1 | SW.. | C . R ... — ... |
| | 20 | 62 | 64 | 59 | $29^7$ | — | — | 3 | 1 | 1 | — | — | S... | R .. F ... R ... |
| | 21 | 58 | 65 | 57 | $29^8$ | 30 | 1 | 12 | 15 | | | | NW.. | F ... |
| | 22 | 59 | 62 | 54 | $29^9$ | 30 | 9 | 15 | 11 | | | | W. | C . R ... C .. |
| | 23 | 53 | 72 | 58 | $30^1$ | 30 | 9 | 10 | 17 | | | | W.. | F .. — .... |
| ○ | 24 | 57 | 63 | 61 | $29^9$ | $^7$ | 10 | — | — | — | 5 | 15 | W.. | C .. R .. — ... |
| | 25 | 64 | 73 | 64 | $29^7$ | — | | | | 27 | 20 | — | SW.. | C .. R .. |
| | 26 | 61 | 73 | 61 | $29^8$ | — | | | | 12 | 15 | 17 | W... | F ... C .. |

Quantity of rain from June 26th to July 26th, one inch $\frac{50}{100}$.

21st and 22d. Thunder, with heavy showers. The mercury in the barometer had, on the 7th, 8th, 9th, 10th, and 11th, a very considerable elevation, and the atmosphere uniformly clear, with light variable winds.

In this interval, catarrhal affections have nearly subsided; and the chronic cough, with difficulty of breathing, has decreased two thirds in the records of the Dispensaries in the western part of the metropolis. Among the diseases believed to be under the influence of some peculiar state of atmosphere, cholera has not been overlooked. Its regular occurrence in the summer and autumnal heats, seem to justify the opinion that it is somehow connected with a high state of temperature. The present season presents no evidence that induces any change in this opinion, for the disease has, within this week, made its usual attacks, and in some instances with considerable severity.

